# ASSESSMENT OF THE QUALITY OF PEDIATRIC CARDIOPULMONARY RESUSCITATION USING THE *IN SITU* MOCK CODE TOOL

**DOI:** 10.1590/1984-0462/2020/38/2018173

**Published:** 2020-01-13

**Authors:** Gabriela de Sio Puetter Kuzma, Camila Bellettini Hirsch, Angélica Luciana Nau, Analiz Marchini Rodrigues, Eduardo Maranhão Gubert, Leonardo Cavadas Costa Soares

**Affiliations:** a Hospital Pequeno Príncipe, Curitiba, PR, Brazil.; b Universidade de São Paulo, São Paulo, SP, Brazil.

**Keywords:** Heart arrest, Cardiopulmonary resuscitation, Simulation training, Pediatrics, Parada cardíaca, Reanimação cardiopulmonar, Treinamento por simulação, Pediatria

## Abstract

**Objective:**

To evaluate the quality of individual and team care for cardiac arrest in a pediatric hospital using clinical surprise simulation (*in situ* mock code).

**Methods:**

We conducted an observational study with a sample of the hospital staff. Clinical simulations of cardiorespiratory arrest were performed in several sectors and work shifts. The mock code occurred in vacant beds of the sector without previous notification to the teams on call. One researcher conducted all mock codes and another evaluated individual and team attendance through a questionnaire contemplating recommendation for adequate cardiopulmonary resuscitation, based on the Pediatric Advanced Life Support (PALS) guidelines. At the end of the simulations, the research team provided a debriefing to the team tested.

**Results:**

Fifteen *in situ* mock code were performed with 56 nursing professionals (including nurses, nursing residents and technicians) and 11 physicians (including two pediatric residents and four residents of pediatric subspecialties). The evaluation showed that 46.7% of the professionals identified cardiac arrest checking for responsiveness (26.7%) and pulse (46.7%); 91.6% requested cardiac monitoring and venous access. In one case (8.3%) the cardiac compression technique was correct in depth and frequency, while 50% performed cardiopulmonary resuscitation correctly regarding the proportion of compressions and ventilation. According to PALS guidelines, the teams had a good performance in the work dynamics.

**Conclusions:**

There was low adherence to the PALS guidelines during cardiac arrest simulations. The quality of cardiopulmonary resuscitation should be improved in many points. We suggest periodical clinical simulations in pediatric services to improve cardiopulmonary resuscitation performance.

## RESUMO

**Objetivo:**

Avaliar a qualidade do atendimento individual e de equipe à parada cardiorrespiratória (PCR) em hospital pediátrico, utilizando a ferramenta de simulação clínica surpresa (*in situ mock code*).

**Métodos:**

Estudo observacional transversal com profissionais de saúde. Foram realizadas simulações clínicas de PCR em diversos setores, em turnos variados, em leito vago do setor sem notificação prévia às equipes de plantão. Um pesquisador conduziu todos os *mock code* e outro avaliou o atendimento individual e de equipe por meio de questionário contendo recomendações para adequada ressuscitação cardiopulmonar baseadas no protocolo do Suporte Avançado de Vida em Pediatria (PALS). Ao término das simulações, realizou-se *debriefing* com a equipe testada.

**Resultados:**

Foram realizados 15 *in situ mock code* e incluídos 56 profissionais de enfermagem (entre enfermeiros, residentes em enfermagem e técnicos) e 11 médicos (sendo dois residentes em pediatria e quatro pediatras residentes em subespecialidade pediátrica). A avaliação mostrou que profissionais identificaram a PCR checando responsividade (26,7%) e pulso (46,7%); 91,6% solicitaram monitorização cardíaca e acesso venoso. Em um caso (8,3%), a técnica de compressão cardíaca foi correta em profundidade e frequência, enquanto 50% executaram a ressuscitação cardiopulmonar corretamente na proporção de compressão e ventilação. As equipes apresentaram bom desempenho na dinâmica de trabalho, segundo variáveis recomendadas pelo PALS.

**Conclusões:**

Houve baixa adesão ao protocolo do PALS durante simulações de PCR, observando-se que a qualidade da ressuscitação cardiopulmonar oferecida pode melhorar em muitos pontos. Sugere-se que, em locais de assistência a pacientes pediátricos, sejam realizados treinamentos com simulações clínicas periódicas para melhor atendimento à PCR pediátrica.

## INTRODUCTION

Successful care for a cardiac arrest event depends on well-trained professionals and adequate resources to enable rapid resuscitation.^1^ Cardiopulmonary resuscitation (CPR) is the only intervention that demonstrates improved chances of survival in a CPA event, a fact that is closely linked to high-quality CPR. High-quality CPR depends not only on individual knowledge, but also on teamwork skills.^2^ Studies evaluating the quality of CPR show that it is not always performed according to the recommendations of international protocols.^3,4^

The *in situ* mock code consists of simulating an emergency situation, such as a CPA, which is performed within a patient care environment. All of the patient resources and facilities are used at a random time unknown by the staff.^5^ The surprise element of the *in situ* mock code allows for the most realistic level of simulation to be attained, bringing in difficulties and emotions that are similar to those encountered when this situation actually occurs.

The aim of this study is to use the mock code methodology to evaluate the quality of individual and team cardiac arrest care in a pediatric hospital.

## METHOD

This was a descriptive, observational, cross-sectional study. The study population consisted of the medical team (including residents), nurses, nursing residents and nursing technicians on duty during the mock code, and those who responded to the emergency call. The study included those medical professionals who, when faced with the fictitious scenario, wanted to participate in the simulation. Those who refused to participate were excluded from the study.

The study was conducted at the Hospital Pequeno Príncipe (HPP), an exclusively pediatric hospital located in southern Brazil (Curitiba, Paraná). The HPP has 32 pediatric medical specialties and 369 hospitalization beds. The hospital is a reference in several pediatric specialties, including for children with a high degree of medical complexity, as well as previously healthy children with community diseases. Fifteen hospitalization sectors were included: the nephrology ward, the general pediatric ward, the neurology ward, the orthopedics ward, the hematology ward, the health insurance pediatric wards, the cardiology ward, the Neonatal Intensive Care Unit (ICU), the Surgical ICU, the General ICU, and the public and private emergency departments.

The mock code occurred during random work shifts (weekdays and weekends; morning, afternoon and evening). A randomized selection was made to define the order of the evaluated sectors. The teams were not informed about the time when the mock code would take place, in order to maintain the surprise factor. The simulations were previously authorized by the sector leader. They informed their staff that a surprise simulation could occur.

In the hospital there is no rapid response team set up solely for this purpose. When there is an emergency alert, the nursing staff of the sector provides initial care and calls the attending physician on duty. If there are any other doctors nearby, they can provide care. Thus, the simulations were performed by the employees who worked in the evaluated sector, and the attending physician was the first to arrive at the site, regardless of their qualification (assistant or resident). In the hospital, there was no prerequisite emergency training such as the Pediatric Advanced Life Support (PALS)^6^ course for employees.

**Figure 1:**
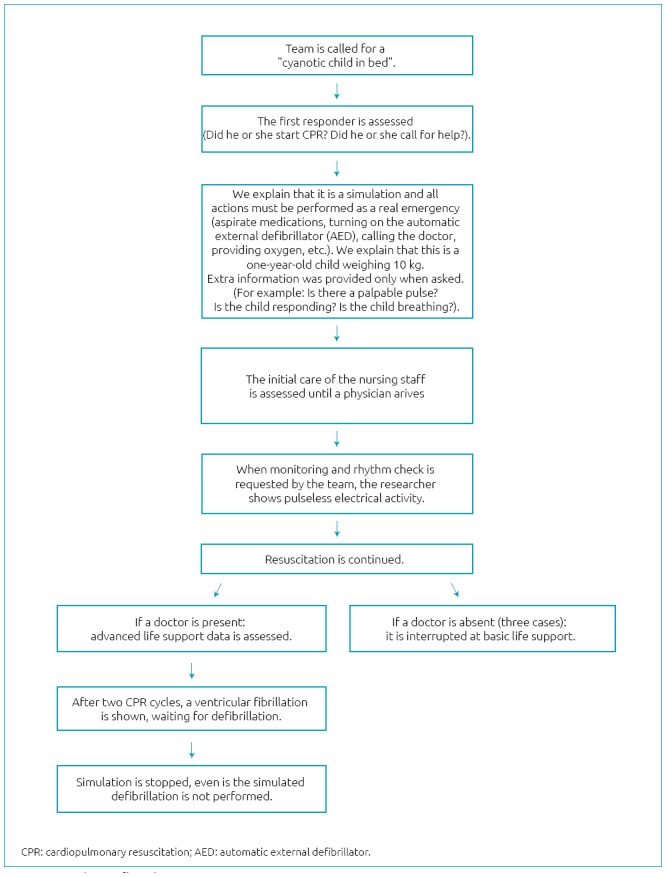
Simulation flowchart.

The *in situ* mock code began with a surprise call in the nursing station, requesting emergency care for a supposed hospitalized patient. A vacant bed of the respective sector was used and a manikin was placed in the bed. Upon the arrival of the first health professional, the following situation was reported: “You got to the bed and realized that this child is cyanotic.” We explained that this is a simulation and that all actions must be performed as a real emergency (aspirate medications, turning on the automatic external defibrillator (AED), calling the doctor, providing oxygen). A researcher stood next to the manikin and provided information when asked. The same case was used in all of the mock codes: a one-year-old child weighing 10 kg, who was unresponsive and had no palpable pulse. When monitoring and rhythm check were requested by the team, the researcher initially showed a pulseless electrical activity (PEA), and after two CPR cycles, a ventricular fibrillation (VF). Even if all the actions were correctly performed, the scenario invariably evolved including all stages of care, and ended after the VF rhythm. The action flowchart is shown in Figure 1.

By the end of the simulation, the research team conducted a debriefing session with the tested team, reviewing the CPR sequence, highlighting the positive points and shedding light on points to be improved.

All the cases were conducted by the same researcher, who is a PALS instructor and a pediatric intensivist. At least one additional PALS-trained researcher was present to observe the care and to complete a questionnaire developed by the research team, which covered all of the items considered to be necessary for adequate cardiac arrest care. The questionnaire consisted of closed-ended questions with the possibility of answering “yes” or “no” and an open question about inadequate medication use during CPR. The two researchers independently assessed all the questionnaire variables. The questionnaire was completed during the simulation. Soon after the simulation, a case summary was made by the researchers to resolve any discrepancies.

The questionnaire was based on PALS recommendations and consisted of four evaluation axes:

- CPR material availability.
- Initial care (identifying cardiac arrest, CPR initiation and technique).
- Advanced care (requesting procedures, CPR technique, drugs).
- Teamwork dynamics.

The following correct resuscitation criteria were considered:

- If no advanced airway, 15:2 compression-ventilation ratio with two rescuers or 30:2 compression-ventilation ratio with one rescuer.
- In the presence of an advanced airway, continuous chest compressions at a rate of 100 to 120 times per minute and one breath every six seconds.
- Chest compressions at a rate of 100 and 120 times per minute, chest compressions at a depth of one third of the anteroposterior diameter of the chest, and chest recoil after each compression.
- Adrenaline at the dose of 0.01 mg/kg or 0.1 mL/kg of 1:10 dilution every three to five minutes.

Correct monitoring was considered when AED or cardiac monitoring with electrodes were used.

Team dynamics were assessed according to PALS criteria:^6^

- **Closed-loop communication:** Orders acknowledged and confirmed when given. Orders announced when executed.
- **Clear messages:** Team members speak clearly. Orders are questioned when doubt exists.
- **Clear roles and responsibilities:** All team members have appropriate roles. Roles are reallocated when appropriate.
- **Knowing One’s Limitations:** Calls for assistance and seeks advice when appropriate.
- **Knowledge Sharing:** Sharing information between team members. Asking for ideas and suggestions.
- **Constructive intervention:** Identifies priorities. Questions colleagues who make mistakes.
- **Reevaluation and Summarizing:** Reevaluates patient. Summarizes patient condition and treatment plan.
- **Mutual respect:** Speaks in a professional, friendly tone of voice. Provides positive feedback.

The variables were described in numbers and percentages. Data analysis was made using Excel software. The study was submitted and approved by the Research Ethics Committee of the hospital. All of the study participants signed a Free and Informed Consent that was applied after the debriefing period.

## RESULTS

Fifteen *in situ* mock codes were performed during different shifts in 15 hospital sectors, which were distributed in ten wards, three intensive care units and two emergency departments, from January to March 2017. In total, 56 nursing professionals (including nurses, nursing residents and nursing technicians) and 11 physicians (two of them pediatric residents and four pediatric residents of a pediatric subspecialty) were evaluated. The same doctor on duty participated in two simulations on different days, and one nurse repeated the simulation when relocated to another sector on a different day from her first simulation.

In all of the cases, emergency carts, orotracheal intubation material, as well as venous access material were available. In one case (6.7%), there was no accessible bag-valve-mask device or rigid CPR board. In two cases (13.3%), there was no defibrillator in the evaluated sector. Data on material availability during the mock code are found in Table 1.

**Table 1:** Availability of materials for cardiopulmonary resuscitation (n = 15).

Material availability	Yes, n (%)
Defibrillator	13 (86.7)
Bag-valve-mask device	14 (93.3)
Rigid board	14 (93.3)
Emergency cart	15 (100)
Orotracheal intubation material	15 (100)
Venous access material	15 (100)

In response to the mock code, nursing professionals were the first responders in 86.7% of the cases. In the other 13.3%, the first approach came from the medical team.

The evaluation of initial care showed that professionals identified cardiac arrest by checking responsiveness (26.7%) and pulse (46.7%). Most called for help (86.7%), while 46.7% started resuscitation. Among those who started CPR, none of them did it correctly in terms of frequency, depth, and ventilation/compression ratio. The initial care assessment is found in Table 2.

**Table 2:** Evaluation of initial care (n=15).

Initial Care	Yes, n (%)
Checking for Responsiveness	4 (26.7)
Checking Pulse	7 (46.7)
Calling for help and using a defibrillator	13 (86.7)
CPR promptly started after identifying cardiac arrest	7 (46.7)
CPR technique correct from the beginning*	0 (0)

CPR: cardiopulmonary resuscitation; *considering frequency, depth and compression/ventilation ratio.

All teams requested emergency carts and supplemental oxygen; 91.6% requested venous access and cardiac monitoring. The rigid board was used by 50% of the teams.

When evaluating advanced life support performance, in one case (8.3%) the CPR technique was correct in depth and frequency, while 50% performed CPR correctly at a 15:2 compression-ventilation ratio with two rescuers. In 91.6% of the cases, orotracheal intubation was requested and 33.3% maintained the correct compression-ventilation ratio.

Regarding medications for CPA management, all of the leaders used adrenaline, although 25% did not perform the dilution and the dosage of 0.01 mg/kg correctly. In 75% of the cases, the frequency of epinephrine administration was correct, and the interval of three to five minutes between doses was respected. In three cases (25%), the leader requested a drug that was inadequate: midazolam and morphine before intubation, and in two times the amiodarone was asked for immediately after the first defibrillation.

At the end of each cycle (two minutes), 33.3% of the teams evaluated rhythm and, if necessary, pulse, and 66.6% changed the rescuer doing the chest compressions. Advanced care data is expressed in Table 3.

**Table 3:** Evaluation of advanced life support (n = 12).

Advanced life support	Yes, n (%)
Emergency cart requested	12 (100)
Cardiac monitoring requested	11 (91.7)
Correctly cardiac monitoring	10 (83.3)
Venous access requested	11 (91.7)
Oxygen requested	12 (100)
Rigid board requested	6 (50.0)
CPR Technique:	
Adequate compression/ventilation ratio	6 (50.0)
Adequate compression rate and depth	1 (8.3)
Orotracheal intubation requested	11 (91.7)
Correct compression/ventilation ratio after intubation	4 (33.3)
Drugs:	
Adrenaline requested	12 (100)
Correct adrenaline dose and dilution	9 (75.0)
Correct frequency of epinephrine administration	9 (75.0)
Inadequate drug application	3 (25.0)
Rhythm and pulse checked when needed	4 (33.3)
Compressors changed every 2 minutes	8 (66.7)

CPR: cardiopulmonary resuscitation.

Team dynamics were also evaluated (Table 4). Most worked with mutual respect (100%), shared their knowledge (100%), knew their limitations (93.3%) and used clear messages (86.7%). In 60% of the emergency care situations, the roles and responsibilities were clear, and in 20%, closed-loop communication was used.

**Table 4:** Team dynamics (n = 15).

Present criteria	n (%)
Closed-loop communication	3 (20.0)
Clear messages	13 (86.7)
Clear roles and responsibilities	9 (60.0)
Knowing one’s Limitations	14 (93.3)
Knowledge sharing	15 (100)
Constructive intervention	14 (93.3)
Reevaluation and Summarizing	9 (60.0)
Mutual respect	15 (100)

Advanced life support data were excluded in three simulations: in one of them, the doctor refused to participate; in the other, a real emergency occurred during the simulation; the last one was stopped because the attending physician took more than ten minutes to reach the scene.

## DISCUSSION

This study assessed cardiac arrest care in a pediatric tertiary hospital using the *in situ* mock code *and* found that its quality is below the recommended international protocols. To our knowledge, this is the first Brazilian study to evaluate the quality of pediatric cardiac arrest care.

Positive outcomes for patients following medical emergencies depend on the ability of the first responders - generally nursing staff - to rapidly perform the necessary care in the first few minutes.^5^ Regarding this, our data showed some problems. When called for a potentially seriously ill child, most professionals did not assess the patient’s responsiveness (73.3%) and pulse (53.3%), delaying cardiac arrest recognition and the start of CPR. In almost half of the simulations (46.7%), CPR was promptly initiated. This result has a direct implication on the patient’s prognosis, since prompt and effective care modifies the cardiac arrest prognosis.^7^ Herlitz et al. evaluated in-hospital cardiac arrest and found that survival to discharge was 33% among patients in whom CPR was started within the first minute as compared with 14% among the patients in whom CPR started more than 1 minute after collapse.^7^

The most alarming part of our study is the poor quality of the chest compression technique performed by the teams. No team performed it correctly in all aspects from the beginning. After the doctor arrived, only one team (8.3%) corrected the technique in all aspects. The remaining (91.7%) maintained ineffective chest compression when frequency and/or depth were evaluated. Only 33.3% checked the rhythm and, when necessary, pulse after each CPR cycle. The quality of CPR is known to directly affect the outcome of cardiac arrest.^4,8^ The resuscitation guidelines set specific recommendations regarding chest compression rate and depth, time to alternate compressors and the need to minimize compression interruptions. The American Heart Association (AHA) considers that effective CPR is more important than drugs and advanced airway for cardiac arrest survival, emphasizing the need of high-quality CPR.^6^ However, as in our study, there is increasing international evidence that CPR quality remains suboptimal. ^3,4,9-16^ Semark et al. evaluated the quality of chest compressions in in-hospital cardiac arrest and found poor compression quality in 96% of the cases when frequency and depth were analyzed.^14^ Abella et al. also studied the quality of in-hospital CPR and found similar data to our findings, showing that the quality of multiple parameters was inconsistent and often did not meet the recommendations of published guidelines, even when performed by well-trained professionals.^16^ Sutton et al. analyzed the quality of in-hospital pediatric CPR, showing that the compressions were shallow in 27.2% of the cases, used excessive residual leaning force in 23.4%, and had an inadequate rate in 43.1%.^9^ Arshid et al. evaluated CPR quality during pediatric resuscitation training. Most sessions had suboptimal CPR performance quality during pediatric resuscitation training, with inadequate chest compression depth in half of the sessions. In addition, team leaders-in-training had little awareness of this inadequacy, even though they were experienced and certified by PALS.^4^ This was also observed in our study, as the compression technique did not improve after the doctor arrived. At this point, the presence of the team leader should be emphasized, because they should guide and coordinate the team, caring for high quality CPR and correcting the technique whenever necessary.

In 50% of the simulations, the ventilation-compression ratio was inadequate. Excessive ventilation should be avoided as it increases intrathoracic pressure and jeopardizes venous return, decreasing cardiac output, coronary perfusion and cerebral blood flow, and increases the risk of regurgitation and aspiration in children without an advanced airway.^17^

Among the reasons for poor quality care, we consider the difficulty of maintaining previously acquired theoretical knowledge. Studies on the retention of CPR skills have shown a pattern of significant reduction in CPR skills within days, weeks, and months after a CPR training.^6^ This emphasizes the need for regular training and effective team dynamics, with feedback and frequent clinical simulations that realistically promote the training environment.^18^ This study encouraged the hospital to provide pediatric emergency training and continuous education for more than 200 professionals, including medical and nursing staff. Another reason for the poor performance in CPR may be the lack of professional confidence, since pediatric cardiac arrest is a rare event; but when it occurs it requires fast, complex and skilled care.^5^

We also evaluated team dynamics, considering the topics recommended by PALS. In this regard, we observed good performance and the presence of mutual respect and knowledge sharing. On the other hand, we did not find much use of closed-loop communication, a strategy that improves the effectiveness of teamwork and reduces errors.^6^ Good team dynamics associated with poor CPR performance might be explained by the teams having previously worked together. Besides, in a real situation there are unexpected events that throw team synchrony off balance.

The clinical mock code is an old and useful tool. AHA recognizes the effectiveness of simulation-based training in enhancing participants knowledge, skills, team performance, leadership and communication.^6^ Our study was conducted as realistic as possible, during the work shift, within the hospital wards and without prior warning. This helped us evaluate teams within their real work dynamics.

The limitations of this study are that data were collected by human observers, who are susceptible to errors. These errors were minimized by the presence of two observers, who simultaneously assessed team performance. All of them had PALS training and one was obligatorily a PALS instructor. Also, the same case being used in all the simulations also represents a limitation, since the first teams could leak the case to the others. Another limitation lies in the fact that the quality of chest compressions was assessed as a whole. Chest compression was considered to be inadequate when it did not attend the recommended rate and/or depth, and did not distinguish between these two criteria. The assessment of care observed during simulations may not correspond to the one offered in real life. However, since cardiac arrest is not a frequent event in most hospital wards, simulated trainings become easier to replicate. Acquiring CPR knowledge and skills is a difficult process. In addition to knowing the theory, the practitioner needs to be secure and have practical skills for quick and effective management. The effectiveness of clinical simulations in learning and improving emergency care performance is well established.^19-22^ By creating a safe and realistic environment, clinical simulation has proven to increase knowledge, practical skills, confidence and emotional control in crisis situations, elements required for good cardiac arrest care.^5,19,22,23^ Another benefit of simulations is their ability to train multidisciplinary teams, help identify human errors, and modify team behavior, which leads to the reduction of errors and improved clinical outcomes.^19,24^ Thus, we stress the importance of continued training with clinical simulations, followed by debriefing sessions with teams, ensuring health professionals continued education. Team leaders must be well trained so that they can recognize and correct the quality of their team’s CPR. Similarly, first responders need to be ready for rapid cardiac arrest PA recognition and the start of CPR.

Our study demonstrates the low level of adherence to PALS protocol during cardiac arrest simulations, making Brazilian data similar to international data. We emphasize the idea that CPR quality can be improved in many ways, especially concerning chest compression technique. In this regard, we suggest periodical training with clinical simulations in pediatric care facilities in order to provide better care for pediatric cardiac arrest.
